# Supplementary material for: Fabrication of PA-PEI-MOF303(Al) by Stepwise Impregnation Layer-by-Layer Growth for Highly Efficient Removal of Ammonia
Source: Nanomaterials (Basel). 2023 Feb 14;13(4):727. doi: 10.3390/nano13040727 (PMC9964625; doi:10.3390/nano13040727)
Supplement: Supplementary file 1 [file nanomaterials-13-00727-s001.zip › nanomaterials-2204318-supplementary.pdf]

Supplementary materials for

Fabrication of PA-PEI-MOF303(Al) by Stepwise Impregnation Layer-by-Layer Growth for High-efficient Removal of Ammonia

Liang Lan[1,2,†], Xuanlin Yang[2,†], Kai Kang[2], Hua Song[2], Yucong Xie[1,2], Shuyuan Zhou [2*], Yun Liang[1], Shupei Bai[2*]

1 School of Light Industry and Engineering, South China University of Technology, Guangzhou, Guangdong 510640, China.

2 State Key Laboratory of NBC Protection for Civilian, Beijing 102205, China.

* Correspondence: E-mail address: baishupei@sklnbcpc.cn (S.B.); zhoushuyuan@sklnbcpc.cn (S.Z.)

† These authors contributed equally to this work.

1. Experimental Section

1.1 Materials.

Aluminum chloride hexahydrate (99%), 3, 5-Pyrazoledicarboxylic acid monohydrate (97%), methyl alcohol (99%) and sodium hydroxide (97%) were purchased from Shanghai Maclean's Reagent Co., LTD. All chemical reagent materials were used directly without pretreatment.

1.2 Synthesis of the PA-PEI-MOF303(Al)

1.2.1 In-situ Growth Method

PA-PEI was placed in a round bottom flask with a pressure equalizing funnel with double PTFE stopcocks attached to the upper end and treated under vacuum at 393 K for 2 h. MOF-303(Al) precursor solution were prepared by using the procedures described in the literatures[68]. 3, 5-pyrazoledicarboxylic acid monohydrate (1.1 g, 0.19 M) was dissolved in deionized water (30 mL), 2.5 mL aqueous NaOH solution (2.57 M) was then added as drops, and the mixture was kept for 30 mins in a pre-heated oven at 393 K under stirring. Then, $AlCl_3·6H_2O$ (1.54 g, 0.19 M) was dissolved in the mixture under sonication. Once a clear solution was formed, it was immediately added to a round bottom flask containing the ligand under vacuum and sonicated for 5 mins. Subsequently, the matrix and ligand solution were all transferred to a teflon-lined autoclave reaction kettle and reacted at 373 K for 24 h. After the system was cooled to room temperature, the composites were washed with methanol using a Soxhlet extraction device for 24 h. In-situ growth of PA-PEI-MOF303(Al) composites were completed after being dried under vacuum at 343 K for 24 h.

1.2.2 The synthesis of PA-PEI-MOF303(Al) with different loadings of MOF303(Al) by Stepwise Impregnation LBL Growth Method:

PA-PEI was placed in a round bottom flask with a pressure equalizing funnel with double PTFE stopcocks attached to the upper end and treated under vacuum at 393 K for 2 h. A 1.08 M metal salt solution was prepared by dissolving 5.2 g aluminum chloride hexahydrate in 20 ml deionized water. Then the metal salt solution was added to the round bottom flask under vacuum and sonicated for 5 mins. After aging for 24 h, the salt solution was filtered out. The PA-PEI-Al modified substrates were obtained by being dried in an oven at 343 K until constant weight. Similarly, the PA-PEI-Al material was vacuum-treated at 393 K for 2 h. 1.1 g of 3, 5-pyrazole dicarboxylic acid monohydrate was dissolved in 30 mL of deionized water, then 3 mL of sodium hydroxide solution (2.57 M) was added and sonicated until the ligand was well dissolved to prepare 0.19 M ligand solution. The ligand solution was then added to a round bottom flask containing a PA-PEI-Al substrate under vacuum and sonicated for 5 mins. Subsequently, the matrix and ligand solution were all transferred to a teflon-lined autoclave reaction kettle and reacted at 373 K for 24 h. After the system was cooled to room temperature, the composites were washed with methanol using a Soxhlet extraction device for 24 h. One-cycle stepwise impregnation LBL growth of PA-PEI-MOF303(Al) composites were completed

after being dried under vacuum at 343 K for 24 h. For the twice-cycle impregnation LBL growth, the prepared PA-PEI-MOF303(Al) composites above were impregnated with metal salt solution and ligand respectively, then regrown in a teflon-lined autoclave. The synthesis conditions were the same as that of one-cycle stepwise impregnation LBL growth method. Five-cycle stepwise impregnation LBL growths of PA-PEI-MOF303(Al) were completed through five times of impregnation and regrowth processes. The above prepared composites are donated as PA-PEI-MOF303(Al)-1-cycle, PA-PEI-MOF303(Al)-2-cycle, and PA-PEI-MOF303(Al)-5-cycle, respectively. The PA-PEI-MOF303(Al) mentioned in the main text is PA-PEI-MOF303(Al)-5-cycle.

The weight percentages of MOF303(Al) on PA-PEI substrate were calculated according to the reported literature [1,2] and the results were summarized in Table S1. The amount of MOF loaded on the substrate can be given as a loading fraction, l_{MOF}, of the total mass of MOF on the substrate per unit mass of the substrate[40]:

$$l_{MOF} = \frac{mass_{MOF-on-Substrate}}{mass_{Substrate}} = \frac{mass_{MOF+Substrate}}{mass_{Substrate}}$$

The MOF wt% is related to loading: wt% = $l_{MOF}\cdot(1+l_{MOF})^{-1}$.

1.3 Microstructure of PA-PEI-MOF303(Al)

The growth states of MOF303(Al) on PA-PEI using in-situ growth and stepwise impregnation LBL growth method were exhibted in SEM characterization (Figure. S2). As shown in Figure. S2, only a few MOF303(Al) grown on the external surface (Figure. S2b) and nearly no MOF303(Al) grown on the internal surface (Figure. S2c) using in-situ growth method compared with PA-PEI substrate (Figure. S2a). This may because the MOF303(Al) nucleation growth rate was higher than the rate at which the precursor solution diffused to the substrate, making it difficult for Al^{3+} to bind to the amine groups on the substrate surfaces. We therefore use the stepwise impregnation LBL growth method, first impregnated the substrate material with an Al^{3+} salt solution to enrich Al^{3+} ions on the substrate surfaces and then added the ligand solution to allow the growth process to progress. MOF303(Al) began to grow on the internal surface of PA-PEI-MOF303(Al)-1-cycle composites (Figure. S2d). With the increase of the number of cycles, the loading amounts of MOF303 significantly increased on the internal surface of PA-PEI-MOF303(Al) (Figure. S2d-3f). After five cycles, the loading amounts remained nearly unchanged. Therefore, stepwise impregnation LBL growth method solved the problems of poor MOF loading using the in-situ growth method by giving a large number of growth sites and achieving a high growth rate on the porous polymer substrate.

2. Supplementary Tables and Figures

Table S1. Tap densities and the weight percentages of MOF303(Al) on PA-PEI substrate prepared by stepwise impregnation layer-by-layer growth method with different number of cycles.

	Tap density/ (g/cm³)	Weight percentage of MOF303(Al) in the composite/ (wt%)
Cycle 1	0.236	27.97
Cycle 2	0.303	42.89
Cycle 5	0.348	51.15

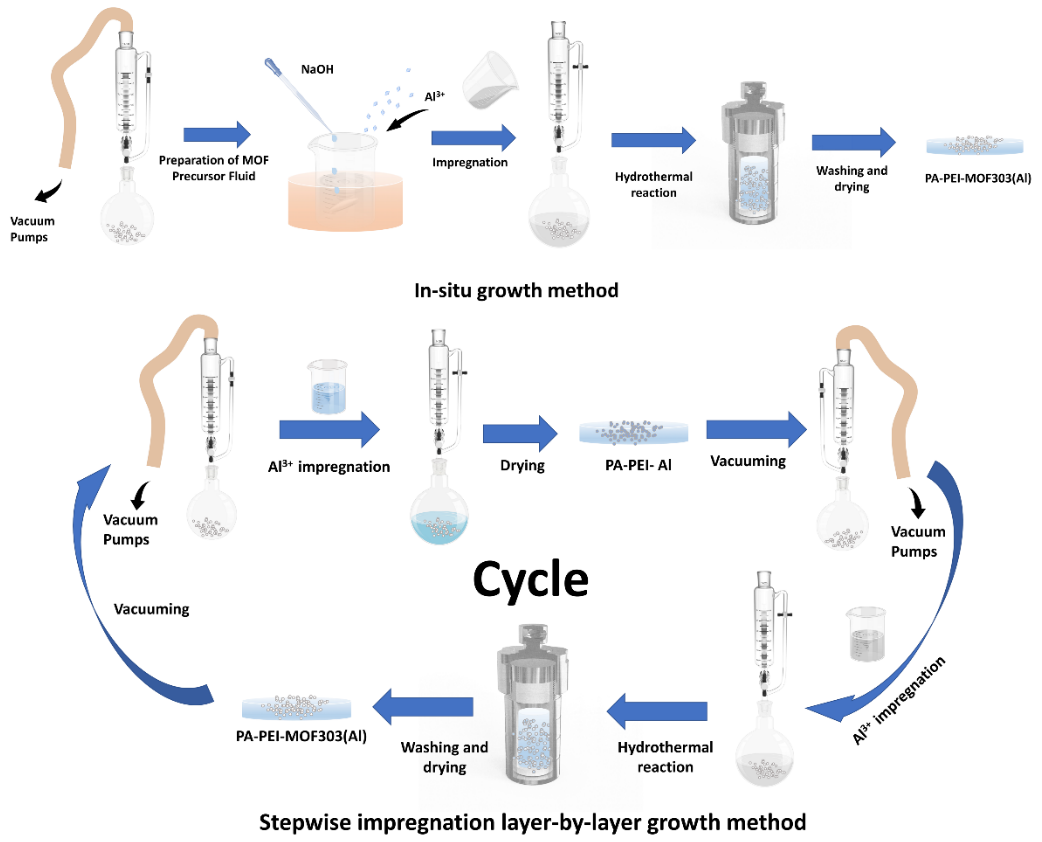

Figure S1. The preparation process of PA-PEI-MOF303(Al) by in-situ growth method and stepwise impregnation layer-by-layer growth method.

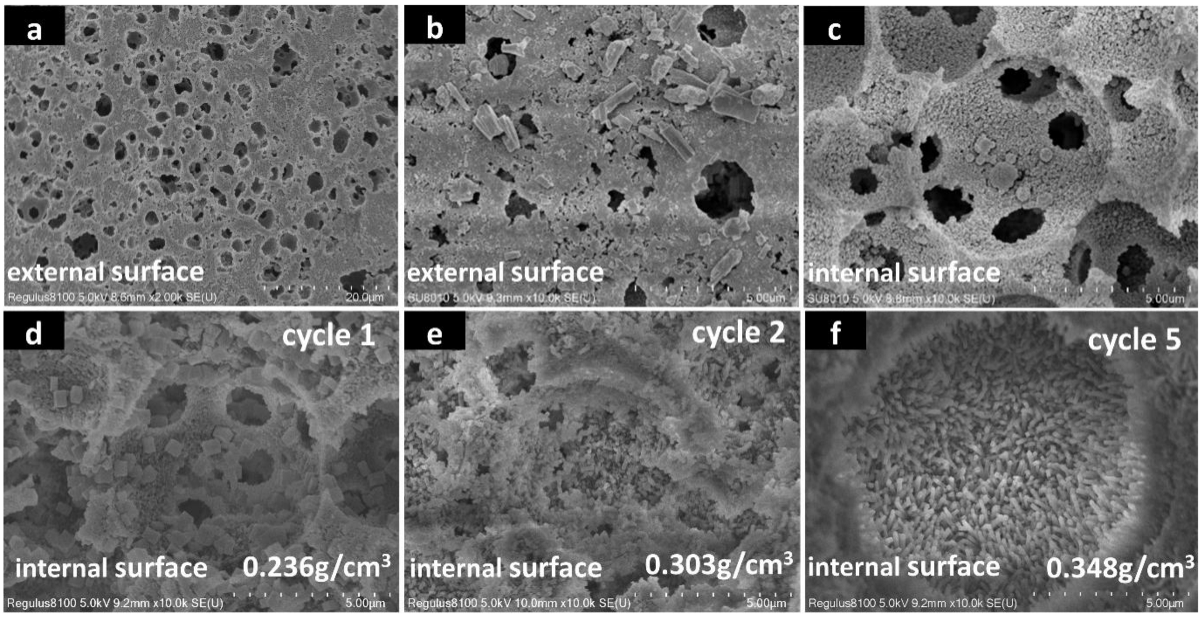

Figure S2. SEM images of (a) PA-PEI, (b) external surface of PA-PEI-MOF303(Al): in-situ growth, (c) internal surface of PA-PEI-MOF303(Al): in-situ growth, and internal surface of (d) PA-PEI-MOF303(Al)-1-cycle, (e)PA-PEI-MOF303(Al)-2-cycle, and (f)PA-PEI-MOF303(Al)-5-cycle.

References

1. Fathieh, F.; Kalmutzki, M.J.; Kapustin, E.A.; Waller, P.J.; Yang, J.; Yaghi, O.M. Practical water production from desert air. *Science Advances* **2018**, *4*, doi:10.1126/sciadv.aat3198.

2. Peterson, G.W.; Lee, D.T.; Barton, H.F.; Epps, T.H.; Parsons, G.N. Fibre-based composites from the integration of metal–organic frameworks and polymers. *Nature Reviews Materials* **2021**, *6*, 605-621, doi:10.1038/s41578-021-00291-2.
